# Can the recent sex-specific evolutions in elite running performances be attributed to advanced footwear technology?

**DOI:** 10.3389/fspor.2024.1386627

**Published:** 2024-05-14

**Authors:** Joel Mason, Laura Starc, Jean-Benoit Morin, Emily L. McClelland, Astrid Zech

**Affiliations:** ^1^ Department of Human Movement Science and Exercise Physiology, Institute for Sport Science, Friedrich Schiller University Jena, Jena, Germany; ^2^ School of Behavioural and Health Sciences, Australian Catholic University, Strathfield, NSW, Australia; ^3^ Inter-University Laboratory of Human Movement Biology, University Jean Monnet Saint-Etienne, Saint-Etienne, France; ^4^ Locomotor Performance Laboratory, Department of Nursing & Health Sciences, Texas Christian University, Fort Worth, TX, United States

**Keywords:** longitudinal bending stiffness, running economy, marathon, sprint, supershoes, sex differences, advanced footwear technology, track and field

## Abstract

Recent improvements in elite running performances across all distances have been largely attributed to the introduction of advanced footwear technology (AFT), which features a curved and stiff plate working synergistically with a new generation of midsole foams demonstrating enhanced resilience and compliance. These recent improvements appear to be considerably more pronounced in women's events, highlighted by improvements in road racing world records by an average of 3.7% (range: 2.6%–5.2%) compared to mean progressions of 1.5% (range: 1.3%–1.9%) in the same men's events. Although there is a growing body of research investigating the mechanisms underpinning running performance enhancements derived from AFT, there remains no explanation for potential sex-based differences in their benefits. We overview the currently available evidence and highlight why the recent direction of AFT research provides a barrier to progress by focusing primarily on male athletes. We subsequently provide our perspective on why women may be benefiting from the new generation of shoes more than men, suggest potential mechanisms leading to hypotheses that need to be further investigated in upcoming studies, and finally propose that factors outside of footwear innovation may have concurrently driven the recently observed performance evolutions.

## Introduction

The recent emergence of advanced footwear technology (AFT) has signalled a new era in the world of running, leading to unprecedented performances and spurring a rapidly developing landscape of research and innovation (1). Shoes featuring AFT differ from previous generations of running footwear through a stiff and curved plate element which is embedded within a thick, curved midsole of foam that is “resilient, compliant and energy-returning” (2). Colloquially known as “supershoes”, footwear featuring AFT was first publicly introduced in 2016 and is now extensively used across all running distances by recreational and elite athletes alike, including most recently in sprint and middle-distance disciplines.

Since the introduction of AFT, there has been a notable shift in global running performances (3–9). Road racing has seen the most striking improvements, with athletes using AFT setting new world records across all distances for both men and women. Bermon et al. (3) found that the world's top 100 runners’ seasonal best times for 10 km, half, and full marathons improved by 0.6%–2.3% since the AFT era began. Additionally, elite marathoners showed an average 0.68% improvement in their times when switching from conventional to AFT shoes (5). The introduction of AFT in track spikes, with a stack height limit of 25 mm and 20 mm for middle-distance and sprint distances respectively (compared to 40 mm for road racing shoes), has also impacted performances. After a period of stagnation in sprinting performances, the introduction of AFT spikes in 2020 led to a marked improvement in the top 100 annual performances across most sprint events (10, 11). Although clear and significant improvements across sprint, middle- and long- distance track events have been observed in the AFT era (11), these findings are notably less consistent across events and typically lower in magnitude than those observed in the road racing events.

### The sex gap in running performances

Despite substantial public and scientific interest in the recalibration of running performances, one key trend has seldomly been discussed: the analyses of annual performance data unanimously indicate that the recent improvements in elite running performances coinciding with the use of AFT are far more pronounced in women than in men across all running distances (3, 4, 7, 9, 10). Specifically, Bermon and colleagues (3) observed that recent improvements in the annual 10 km, half marathon and marathon performances of the world's best athletes were between 1.7% and 2.3% in women compared to 0.6%–1.5% for men. Further, Senefeld et al. (4) found that in elite marathon runners, AFT provided double the benefit for women than it did for men, corresponding to improvements of 1.6% and 0.8%, or 3.7 min and 1.2 min, respectively. These sex-specific improvements are not just restricted to road running. While recent improvements in the annual performances of elite men's sprint athletes have ranged between 0.40% and 0.98% since the dawn of the AFT era, the performances of the top female athletes have improved by up to 1.52% (10), and women's track events of all distances have improved more consistently than men's events (11).

**Figure 1:**
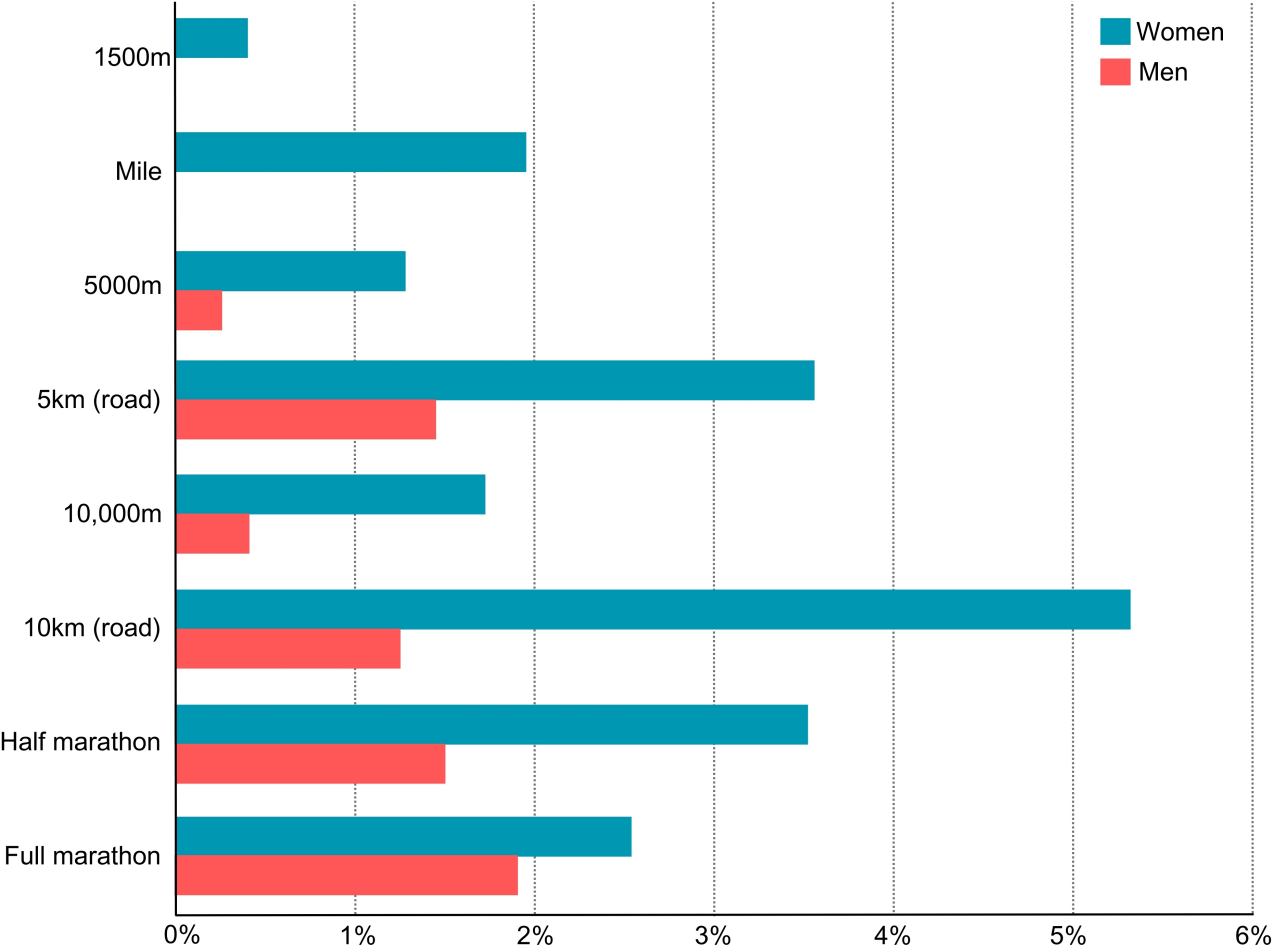
Percentage improvement in world record performances of middle- and long-distance events for men and women since the introduction of advanced footwear technology.

**Figure 2:**
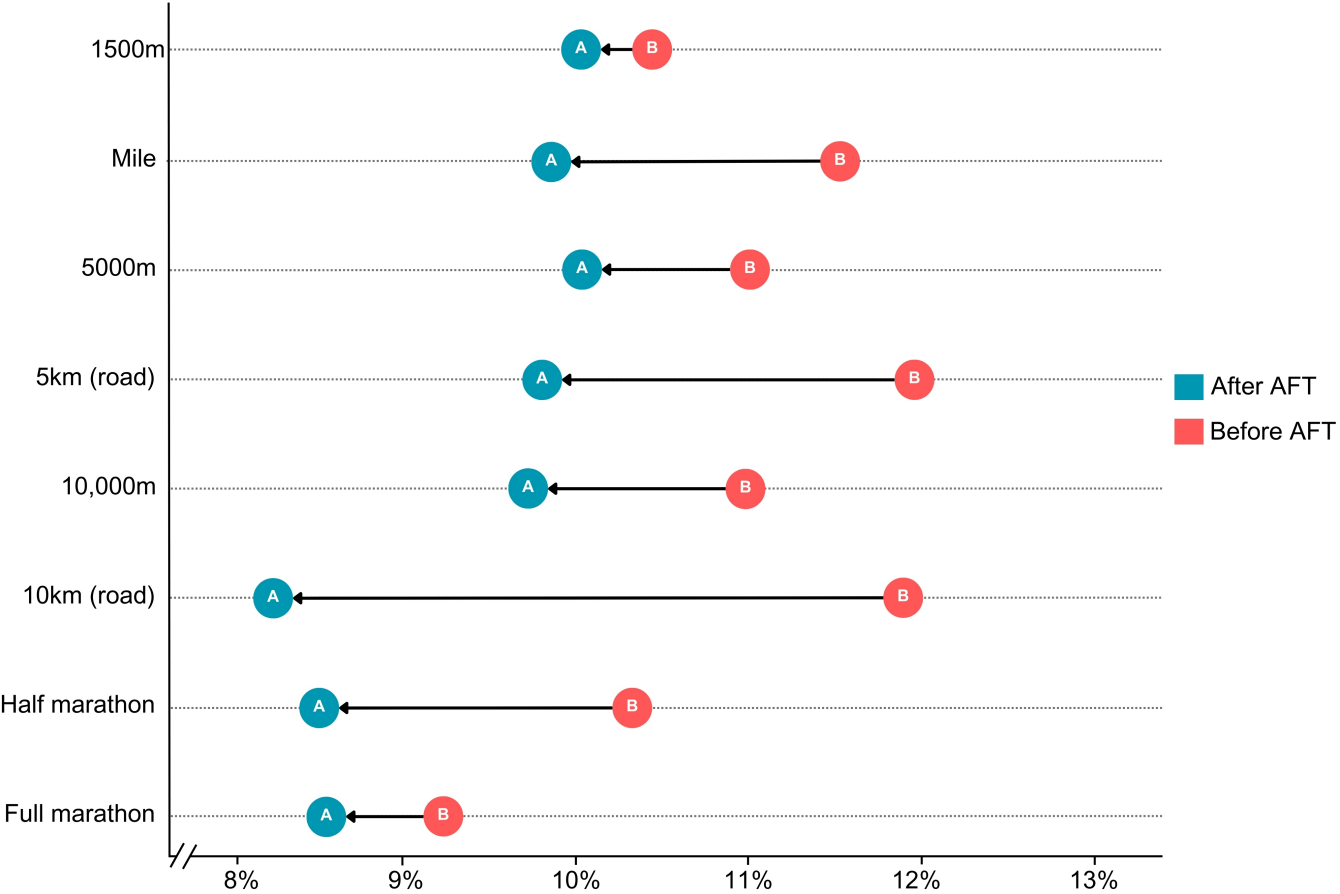
The sex gap in world record performances across middle- and long-distance running events before and after the introduction of advanced footwear technology.

Overall, the latest sex-specific improvements in performance are perhaps best demonstrated by (1) the recent progressions in world records for men vs. women (Figure 1), and (2) the associated narrowing of the sex gap in world record performances. Traditionally, this sex gap over road running distances is around 12% (12) but has recently narrowed significantly for every event from 1,500 m onwards, dropping to 9% or lower across 10 km, half marathon and marathon distances (Figure 2).

### Mechanisms of AFT benefits

Although there are several possible reasons for these performance improvements, evidence suggests that AFT is a key driver (3–11). Understanding why women may benefit more from AFT than men first requires an understanding of the mechanisms through which the elements of AFT generally create performance advantages [for review, see (13, 14)]. Improvements in running economy at submaximal velocities (15) seem to arise from the combination of a) an increase in longitudinal bending stiffness (LBS), which limits energy dissipated at the metatarsophalangeal (MTP) and ankle joints during dorsiflexion, and b) the new midsole materials, which have a higher energy retention capacity (15, 16). The addition of AFT into track spikes and its effects on sprint and middle-distance performance metrics remains largely unexplored (17). Performance benefits may be somewhat independent from shifts in running economy and could be due to stiffening of the MTP joints (18), a reorientation of the point where force is applied to facilitate horizontal projection and propulsion (19–22), and/or extensions in leg length (10).

Despite growing evidence, the biomechanical explanations underlying performance enhancements through AFT across all distances remain incomplete (23–25), which is further complicated by the large variations in individual running economy responses to footwear featuring AFT (26, 27). Given that the magnitude of performance improvements coinciding with the release of AFT is far greater in female than male athletes, studies reporting data independently for each sex and specifically studies reporting data in females will not only help explain this phenomenon but could also further uncover the elusive mechanisms of action and mediating factors underpinning AFT. However, the currently available literature appears to take a different approach, with recent reviews indicating that the supershoe research landscape is dominated by male participants.

### The (under) representation of women in AFT research

In a 2022 meta-analysis investigating the effects of LBS on running economy and ground contact biomechanics, women were excluded “due to the low number compared to the total number of men in the whole sample” (28). More recently, Knopp and colleagues (26) conducted a meta-analysis characterising the variability of responses to AFT but were only able to identify one study with female participants. Further compelling evidence comes from a recent review characterising the influence of general footwear features on the metabolic cost of running (29). Across LBS, ‘feeling of comfort’ and the shoe cushioning system, only 33 of 257 pooled participants were women, consisting just 12.84%.

To corroborate these findings, we performed a simple reverse search of the literature by screening the abstracts of all studies which have cited the original seminal AFT paper by Hoogkamer and colleagues (15). Papers were identified using Google Scholar's ‘cited by’ tool. To uncover any further relevant papers, we performed an additional systematic search of Google Scholar and PubMed databases using the keywords “longitudinal bending stiffness”, “supershoes”, “advanced footwear technology”, “midsole”, “footwear” + “running economy”, “energetics”, “biomechanics” and “metabolic”, in line with previous reviews on the topic (26, 28). All peer-reviewed articles published in English since 2018 (and until April 1 2024) were considered, and articles titles and abstracts were screened by a single author (JM) using Rayyan (30). Papers were deemed eligible for inclusion if they provided primary experimental data regarding at least one element of AFT, such as an increase in longitudinal bending stiffness, use of innovative midsole foam materials or an increase in midsole thickness or rocker profile, and their influence on running performance, biomechanics, or energetics. Analyses of annual performance data were excluded due to not recruiting participants.

51 peer-reviewed experimental studies with available primary data related to AFT were identified, totalling 741 participants, of which 108 were women, constituting just 14.57% of the total sample. Of those 51 studies, 34 exclusively included male participants, 13 encompassed both male and female subjects but rarely disaggregated their data by sex, and just two studies focused solely on female subjects (with two studies not specifying the participants’ sex). While we acknowledge that this screening approach falls short of the rigorous PRISMA literature search guidelines, the intention of this commentary is not to be exhaustive, but to be indicative. Importantly, these rates of female inclusion in AFT research are strikingly below the female participant rates across sport science and sport medicine research in general, which are already at low levels of 34%–39% (31, 32).

There are several potential explanations for why the AFT research landscape is unequally distributed. Available prototype shoes are often limited in size availability, which restricts recruitment of a representative sample. Shoe size is often listed as a recruitment strategy or inclusion criteria (33, 34) which is conceivably linked to research funding. Scientific progress also often depends on homogenous, not always representative, samples to precisely determine the effects of an intervention or condition (35). However, we argue that these factors do not entirely explain the imbalance in sex-representation in AFT research over the last six years. For example, there are multiple publications which elected to analyse males only despite using freely available race data that may be equally accessible for both sexes (5, 6, 36). While research in AFT exclusively focusing on male data might be seen as recognition of sex-related variables influencing outcomes, the scarcity of equivalent studies in females represents a major (and familiar) barrier to further insight, innovation and progress.

### What does the current evidence suggest, and what should future research address?

Notably, the only studies which have reported female-specific data regarding the influence of AFT on running economy indicate that there is no significant sex-based advantage (37, 38). Specifically, Barnes and Kilding (37) reported running economy benefits of between 1.7% and 7.2% when highly trained runners used AFT, however differences between males and females were trivial to small across conditions. Most recently, Martinez and colleagues (38) reported metabolic power improvements of 4.2% and running economy improvements of 3.9% when trained female runners ran in AFT compared to control shoes, which is consistent with findings in males (15). Contrary to these initial findings, women have unique running injury profiles (39), specific running biomechanics (40), as well as unique energetics and fatigue dynamics during running when compared to men (41, 42). Therefore, if AFT truly offers unique performance advantage to female athletes, it is likely grounded in the physiological, biomechanical, and morphological differences between sexes and remains currently undetected due to the insufficiency of research.

Future prospective and observational studies should use the same methods for both sexes and disaggregate data by sex to replicate and extend the above findings using larger sample sizes across all determinants of running performance, particularly given the high degree of variability in running economy responses. Studies should also clarify whether potential unique benefits in women compared to men can be explained by underlying mechanisms or factors. We suggest that the following factors deserve further consideration:

#### Body mass

Sex-based differences in body mass, which average 22.5% in elite track and field runners (43), may influence the energy storage and return elements of AFT. Current evidence is inconclusive, indicating no relationship between body mass and changes in running economy when shoe LBS is increased (44, 45), but an influence on sprint acceleration (46). However, considering findings that increasing LBS alone may be insufficient for improving running economy (47), how body mass interacts with the pairing of increased LBS, the latest generation of midsole materials and updated shoe geometry warrants further investigation across all velocities and distances.

#### Shoe size

If midsole thickness is maintained or at least not scaled proportionally between shoe sizes, the geometry of the shoe is also unique to each size, including a more pronounced rocker profile. Smaller shoe sizes may alter the timing and location of force application or influence the rigidity of the stiffening elements of the shoe. Such changes may influence the energy return from the footwear (48), alter ankle joint mechanics (16, 49), and/or reorient the direction of force application to facilitate horizontal propulsion.

#### Leg length

Given that female runners are typically 6% smaller in stature than their male counterparts (40), similar absolute midsole stack height increases in AFT compared to traditional footwear also create greater relative increases in effective leg length for female athletes and therefore greater increases in relative step length, as we recently proposed (10). This notion is preliminarily supported by evidence demonstrating an increase in stride length when female runners use AFT at submaximal speeds (50).

#### Stride frequency

Women have a higher stride frequency than men across all running distances, and the corresponding higher volume of ground contacts required to cover a given distance compared to men may simply provide more opportunity for the supershoe mechanisms to interact with the ground, potentially compounding the benefits of AFT.

#### Muscle-tendon unit properties

The elements of AFT have been demonstrated to alter fascicle and tendon activity of the gastrocnemius medialis and facilitate propulsion during running (51), which may be influenced by sex (52). Given the comparatively large energy contributions of these endogenous structures relative to the MTP joints and the exogenous energy contributions of footwear, this should not be overlooked.

Whether these innate differences between sexes are enough to infer the physiological advantages which ultimately culminate in the apparently greater performance benefits from AFT for women compared to men remains unclear. To answer this question, one viable approach is for future studies to scale elements of the shoe relative to body mass, leg length and shoe size, and assess subsequent outcomes in running performance and its determinants across various velocities and distances. Further, because the original studies successfully predicting improvements in running performance via lab-based improvements in running economy did so using exclusively male participants (53), future work should address whether there are any sex-based differences in the magnitude of transfer from lab-based changes to ecological running performance changes.

Finally, it should also be considered that even if the identified performance and mechanistic advantages for women compared to men are minor, they are not just available acutely on competition day, but also through the daily use of AFT during training, which may then ultimately compound to result in the larger enhancements seen on race day.

### Which factors beyond AFT which might explain why women have recently improved more than men?

If upcoming studies also fail to reveal sex-based advantages of AFT in the key determinants of endurance and sprint running performance, then potential explanations for the accelerated evolution of recent women's running performances compared to men must be sought elsewhere.

Recent upgrades in co-operative drafting strategies during racing (54) likely benefit women more than men over road racing distances given that they can be paced for the entirety of the race as opposed to only an initial portion, although this does not explain why women are also improving at faster rates on the track. Another consideration is that modern developments in training design and nutritional supplementation strategies which more precisely target female physiology may have also contributed to the latest breakthroughs in performances (55, 56).

An additional possibility is that women are further from their physiological limits than men due to historical inequalities in access, funding and opportunity (57), and that recent improvements in these social factors may have accelerated their modern progress. However, there is evidence indicating that the sex gap in sports performance had not evolved since 1983 (58), and that women's running performances have largely plateaued for the decades most recently preceding the introduction of AFT (59, 60), despite substantial improvements in women's participation, support and access to sport across this period. One important counterpoint is that the evolution of women's performances across the decades has been tarnished by historical performance enhancing drug use, and therefore the true trajectory of performances and sex-specific proximity to physiological limits remains obscured.

A discussion regarding the evolution of performances in running events would be incomplete without further exploration of the inevitable influence of performance enhancing drugs. Across the 2008–2016 Olympiads, 30.6% of medals in running distances from 800 m onwards were won by athletes who had either personally served a doping suspension or had been coached by somebody who has served a doping suspension (61). A further 55.6% of these medals were won by athletes hailing from countries who are either on the world anti-doping authority non-compliant list or watch list, or whose doping control laboratory has had its accreditation suspended (61). Current anti-doping strategies are widely considered insufficient to curtail the use of performance enhancing drugs in track and field (62), and this issue may have been recently exacerbated by large reductions in drug testing worldwide during the pandemic (63), newer approaches to micro-dosing (64) and novel pharmacological agents.

Of particular relevance is analysis of French anti-doping data between 2013 and 2019 which revealed that only 22% of all tests were conducted in women, which does not adequately account for the testing pool which comprises 39% women (65). Importantly, a higher prevalence of blood doping was observed in female (22%), compared to male track and field athletes (15%) at the 2011 World Championships (66). Outside of blood doping, differences in pre-existing levels of circulating hormones may influence the sex- specific benefits received from the use of performance enhancing drugs, including anabolic agents. For example, the notorious former German Democratic Republic's widespread doping program placed special emphasis on administering androgens to women and adolescent girls because this proved especially effective for performance enhancement (67). Overall, the intensified effectiveness of doping in women is demonstrated by the record books: of the 13 current outdoor track and field world records which pre-date the introduction of randomised drug testing, 11 are in women's events. While it is possible that the performance benefits which are commonly attributed to AFT may be concurrently aided by developments in performance enhancing drug use for both men and women, we note that this remains purely speculative.

## Conclusion

Recent rapid developments in racing footwear technology have occurred at a much faster rate than the related academic publishing (25), which is likely to inhibit the sharing of knowledge to stakeholders outside of the brands themselves. Given the lucrative results of the arms race that has evolved between footwear developers, paired with the evidence outlaid in this commentary, it would seem unlikely that companies have not directly researched sex-specific mechanisms and performance benefits of AFT at least internally to maximise their competitive advantages. However, the currently *available* evidence suggests that advancements in footwear technology cannot explain why women's running performance improvements have surpassed those of men in recent years. To challenge this understanding, a substantial shift in research representation trajectory is essential.

## Data Availability

Publicly available datasets were analyzed in this study. This data can be found here: https://worldathletics.org/records/toplists.
